# Dexamethasone-Induced FKBP51 Expression in CD4^+^ T-Lymphocytes Is Uniquely Associated With Worse Asthma Control in Obese Children With Asthma

**DOI:** 10.3389/fimmu.2021.744782

**Published:** 2021-10-15

**Authors:** Vickram Tejwani, Amanda McCormack, Karthik Suresh, Han Woo, Ningchun Xu, Meghan F. Davis, Emily Brigham, Nadia N. Hansel, Meredith C. McCormack, Franco R. D’Alessio

**Affiliations:** ^1^Johns Hopkins University, Division of Pulmonary and Critical Care Medicine, Baltimore, MD, United States; ^2^Cleveland Clinic, Respiratory Institute, Cleveland, OH, United States; ^3^Department of Environmental Health and Engineering, Johns Hopkins Bloomberg School of Public Health, Baltimore, MD, United States; ^4^Flow Cytometry Core, Johns Hopkins University, Baltimore, MD, United States; ^5^Department of Molecular and Comparative Pathobiology, Johns Hopkins School of Medicine, Baltimore, MD, United States; ^6^Division of Infectious Diseases, Johns Hopkins School of Medicine, Baltimore, MD, United States

**Keywords:** asthma, immune mechanism, obesity, steroid-resistance, T-lymphocytes

## Abstract

**Introduction:**

There is evidence that obesity, a risk factor for asthma severity and morbidity, has a unique asthma phenotype which is less atopic and less responsive to inhaled corticosteroids (ICS). Peripheral blood mononuclear cells (PBMC) are important to the immunologic pathways of obese asthma and steroid resistance. However, the cellular source associated with steroid resistance has remained elusive. We compared the lymphocyte landscape among obese children with asthma to matched normal weight children with asthma and assessed relationship to asthma control.

**Methods:**

High-dimensional flow cytometry of PBMC at baseline and after dexamethasone stimulation was performed to characterize lymphocyte subpopulations, T-lymphocyte polarization, proliferation (Ki-67+), and expression of the steroid-responsive protein FK506-binding protein 51 (FKBP51). T-lymphocyte populations were compared between obese and normal-weight participants, and an unbiased, unsupervised clustering analysis was performed. Differentially expressed clusters were compared with asthma control, adjusted for ICS and exhaled nitric oxide.

**Results:**

In the obese population, there was an increased cluster of CD4^+^ T-lymphocytes expressing Ki-67 and FKBP51 at baseline and CD4^+^ T-lymphocytes expressing FKBP51 after dexamethasone stimulation. CD4^+^ Ki-67 and FKBP51 expression at baseline showed no association with asthma control. Dexamethasone-induced CD4^+^ FKBP51 expression was associated with worse asthma control in obese participants with asthma. FKBP51 expression in CD8^+^ T cells and CD19^+^ B cells did not differ among groups, nor did polarization profiles for Th1, Th2, Th9, or Th17 percentage.

**Discussion:**

Dexamethasone-induced CD4^+^ FKBP51 expression is uniquely associated with worse asthma control in obese children with asthma and may underlie the corticosteroid resistance observed in this population.

## Introduction

Asthma is the most common chronic disease in children, and currently, more than 30% of children are overweight or obese (1). Obesity is not only a major risk factor for asthma but a comorbidity that is associated with increased disease severity and worse quality of life. There is mounting evidence that the unique, obese asthma phenotype is less likely to respond clinically to inhaled corticosteroids (ICS), the current first-line therapy for asthma (2–4). Improving our understanding of the mechanisms that underlie obese asthma has important clinical and population implications. Emerging evidence demonstrates peripheral blood mononuclear cells (PBMCs) play an important role in the immunologic pathways of obese asthma and steroid resistance (5, 6). The PBMC bulk transcriptional expression of FK506-binding protein 51 (FKBP51) has been shown to be associated with reduced steroid responsiveness; however, participant’s body mass index (BMI), cellular source, and protein expression for FKBP51 have not been assessed (7). FKBP51 is an intracellular protein classified as an immunophilin given its tight binding to the immunosuppressants FK506 and rapamycin. The protein is encoded by the *FKBP5* gene, which was shown to have increased expression in those with severe asthma in the U-BIOPRED cohort (8). The expression of FKBP51 in peripheral blood cells is known to be induced by corticosteroids (9) and its overexpression can inhibit corticosteroid signaling leading to resistance *via* a negative feedback loop (10, 11). The Ki-67 protein is present in proliferating cells and has previously been used to assess proliferation of different immune cells (12, 13). We sought to characterize peripheral blood lymphocyte signatures, identify the cellular sources of protein FKBP51 expression in obese compared with normal weight children with asthma, and evaluate the relationship between FKBP51 expression and asthma morbidity.

## Methods

### AIRWEIGHS Cohort and Clinical Phenotyping

The AIRWEIGHS study was designed to investigate effects of indoor PM_2.5_ and PM_10_ reduction on asthma morbidity in obese versus normal weight inner-city children with asthma. This randomized, sham-controlled trial of a portable in-home air purifier intervention enrolled a 1:1 ratio of overweight/obese:normal weight children to examine proposed differential effects of the intervention by obesity status. Inclusion criteria were as follows: 8–17 years old, never-smoker, physician-diagnosed asthma categorized as persistent by National Asthma Education and Prevention Program criteria (14), and history of at least one exacerbation in the preceding year. Clinic visits were rescheduled if a participant was acutely ill such that biospecimens would not have been collected in the setting of an active respiratory infection. Serum IgE was obtained four weeks prior to the preintervention visit.

At the preintervention visit, participants provided a blood sample for isolation of PBMC. In addition, they were queried regarding medication use. They also performed lung function testing including spirometry for postbronchodilator forced expiratory volume in 1 s (FEV1) using PiKo meters (nSpire Health, Longmont, CO, USA), and fractional exhaled nitric oxide (FENO) was measured (NIOX TM System, Aerocrine, Sweden). They also completed validated questionnaires to assess asthma morbidity and control: the Asthma Control Test (ACT), Asthma Therapy Assessment Questionnaire (ATAQ), and Asthma Symptom Utility Index (ASUI). The ACT consists of five items, is scored from 5 (poorly controlled) to 25 (complete control) with a score of ≤19 indicating uncontrolled asthma, and has a minimal clinically important difference (MCID) of 3 (15, 16); the ATAQ consists of four items, ranges from 0 (no control problems) to 4 (four control problems) with a score of >0 indicating suboptimal asthma control, and has an MCID of 1 (17); and the ASUI consists 10 items, the score ranges from 0 (poorly controlled) to 1 (well controlled) and has an MCID of 0.09 (18, 19).

PBMCs were obtained and clinical phenotyping performed as described at the prerandomization visit so were uninfluenced by the trial of air purifier intervention. From the total cohort of 160 participants (two underweight, 59 normal weight, 24 overweight, 75 obese), we selected 20 normal weight and 20 obese participants matched for inhaled corticosteroid use, ACT score, and postbronchodilator FEV1% predicted (FEV1pp) after excluding those taking systemic steroids in the last 30 days or on biologics. Normal weight was defined as BMI percentile between 5th and 85th percentiles and obese as BMI≥ 95th percentile (20).

### Peripheral Blood Mononuclear Cell Isolation and Flow Cytometry

We isolated PBMCs using the Ficoll-Paque centrifugation method (Amersham) from normal weight and obese children with asthma enrolled in the AIRWEIGHS cohort. Cells were incubated in serum-free CTL media with human T-activator CD3/CD28 (DynaBeads) for 24 h at 37°C in a humidified 5% CO_2_ atmosphere. GolgiStop and GolgiPlug were added for the final 4 h. For the steroid-responsive protein, FKBP51, expression was also measured after incubation with dexamethasone (DEX) 10^−6^ for 24 h. This dose was determined based on a prior publication (5) and also after dose titration, that it upregulated FKBP51 in pilot studies of PBMCs from a healthy volunteer (**Supplementary Figure S1**). After incubation, samples were stained with a UV excitable LIVE/DEAD (Invitrogen, Waltham, MA, USA) discriminator and then treated with human IgG (Rockland, Pottstown, PA, USA) to block Fc receptors. Cells then underwent surface staining and then were fixed and permeabilized, followed by intracellular staining (**Supplementary Table S1**). Acquisition is performed using a FACSAria instrument with FACSDiva software (BD Biosciences, San Jose, CA, USA) and FlowJo version 10.5.0 (Tree Star, Inc., Ashland, OR, USA).

Single cells were identified followed by live-dead discrimination. Live cells were excluded for CD14^+^ populations and inclusion of CD3^+^ populations to remove monocytes and identify T-lymphocytes, respectively. CD4^+^ and CD8^+^ populations were identified. Within the CD4^+^ population, Th1 cells were defined as IFN-γ^+^, Th2 as IL-4^+^, Th9 as IL-9^+^, and Th17 as IL-17^+^. Regulatory T cells were defined as CD4^+^CD25^+^CD127^−^FoxP3^+^ (**Supplementary Figure S2**). Intracellular TNF-α, FKBP51, and Ki-67 expression were also measured and quantified as geometric mean fluorescent intensity. Fluorescence minus one was utilized to determine specificity for intracellular cytokine and protein expression. Data were analyzed using either FlowJo (Tree Star, Inc.) for traditional gating analyses or R/Bioconductor as previously described (21).

### Statistical Analysis

Given we did not assume normal distribution, two-tailed nonparametric (Wilcoxon) were used to compare differences of cell proportions between obese and normal weight children with asthma. Following this, unsupervised clustering analysis was conducted using FlowSOM and flowCore packages in R/Bioconductor as previously described to identify distinct subpopulations that were increased (by a factor of at least twofold) in either group (21, 22). Linear regression analyses were run to evaluate the relationship between predefined T-lymphocyte subsets and differential clusters to the primary outcome of ATAQ score and secondary outcomes of ACT score, ASUI, and FEV1pp. Models were adjusted for exhaled nitric oxide (eNO) and ICS use, defined as potential confounders based on their bivariate associations (*p* < 0.2) with predefined T-lymphocyte subsets and any measured outcomes. Age, gender, home smoking, and eosinophil count were tested but did not meet criteria for inclusion as covariates. Interaction terms were constructed between obesity status and immune clusters to assess effect modification by obesity status and subgroup analysis performed for obese and normal weight participants to ascertain unique relationships to asthma morbidity in the normal weight versus obese asthma population. Analyses were performed with R version 3.6.3. Statistical significance was defined as *p* < 0.05 for main effects and *p* < 0.10 for interactions.

## Results

The children had a mean [standard deviation (SD)] age of 11.4 years (2.5), were predominantly African American (90%), and most (75%) reported ICS use (**Table 1**). Other than BMI, characteristics were similar in both groups, except nonsignificant trends of higher FVC, lower eosinophil count, and lower FeNO in the obese group. There were no differences between obese versus normal weight in percentage of CD4^+^, CD8^+^, and CD4:CD8 ratio. There were also no differences in percentages of Th1, Th2, Th9, Th17, or regulatory T cells. In addition, adjusted models did not demonstrate an association of these cell population percentages to ATAQ score or other secondary outcomes of asthma control and FEV1pp.

**Table 1:** Participant characteristics.

	All participants (*n* = 40)	Obese (*n* = 20)	Nonobese (*n* = 20)
Gender (number female, %)	16 (40%)	8 (40%)	8 (40%)
Age (years, mean ± SD)	11.4 ± 2.5	11.2 ± 2.2	11.6 ± 2.8
Race (number Black, %)	36 (90%)	18 (90%)	18 (90%)
FEV1 (post-BD % predicted, mean ± SD)^†^	99.2 ± 13.7	101.8 ± 10.1	96.6 ± 16.4
FVC (post-BD % predicted, mean ± SD)	102.0 ± 12.2	105.8 ± 9.7	98.3 ± 13.6
FEV1/FVC % (mean ± SD)	85.2% ± 7.5	84.3% ± 7.3	86.1% ± 7.6
FEF25-75% (post-BD % predicted, mean ± SD)	94.4 ± 28.0	96.0 ± 24.8	92.8 ± 31.5
BMI (percentile, mean ± SD)^*^	73.9 ± 31.6	98.5 ± 1.3	49.3 ± 27.7
BMI (kg/m^2^, mean ± SD)^*^	24.7 ± 8.1	31.3 ± 6.2	18.1 ± 2.4
ICS use in last 2 weeks (number, %)^†^	30 (75%)	15 (75%)	15 (75%)
ACT score (mean ± SD)^†^	19.2 ± 3.6	19.3 ± 4.1	19.1 ± 3.1
ATAQ score (mean ± SD)	2.7 ± 1.9	2.9 ± 2.1	2.6 ± 1.9
ASUI score (mean ± SD)	0.83 ± 0.12	0.86 ± 0.10	0.79 ± 0.14
Eosinophil number (cells/mcL)	327 ± 267	279 ± 249	374 ± 282
Exhaled nitric oxide	32.7 ± 30.9	31.3 ± 22.4	34.2 ± 37.8
Serum IgE	324.18 ± 506.12	253.09 ± 384.85	399.01 ± 610.66

^*^p < 0.05.

^†^Matched on these three variables.

Unsupervised clustering analysis identified a cluster of a CD4^+^ T-lymphocyte subset-expressing FKBP51 and Ki-67 that was more prevalent in obese participants with asthma (2.6% of CD3^+^ T-lymphocytes) compared with normal weight participants (1.1% of CD3^+^ T-lymphocytes, **Supplementary Figure S3**). There was a correlation of FKBP51 and Ki-67 expression in CD4^+^ T-lymphocytes among both obese (*r* = 0.75, *p* < 0.01) and nonobese (*r* = 0.67, *p* = <0.01) participants with asthma. Expression of FKBP51 was then measured in CD4^+^ T-lymphocytes (**Supplementary Figure S4**) and across CD4^+^ T-lymphocyte subpopulations (**Supplementary Figure S5**). There were no statistically significant differences across subpopulations; however, there was a trend (*p* = 0.07) toward higher FKBP51 expression among regulatory T cells in obese participants with asthma. Unsupervised clustering analysis of cells after DEX exposure identified a CD4^+^ T-lymphocyte subset expressing FKBP51 that was more prevalent in obese participants (1.2% of CD3^+^ T-lymphocytes) compared with normal-weight participants (0.5% of CD3^+^ T-lymphocytes, **Supplementary Figure S6**). Expression of DEX-induced FKBP51 was then measured in CD4^+^ T-lymphocytes (**Supplementary Figure S7**) and across CD4^+^ T-lymphocyte subpopulations (**Supplementary Figure S8**). There were no statistically significant differences across subpopulations.

**Figure 1:**
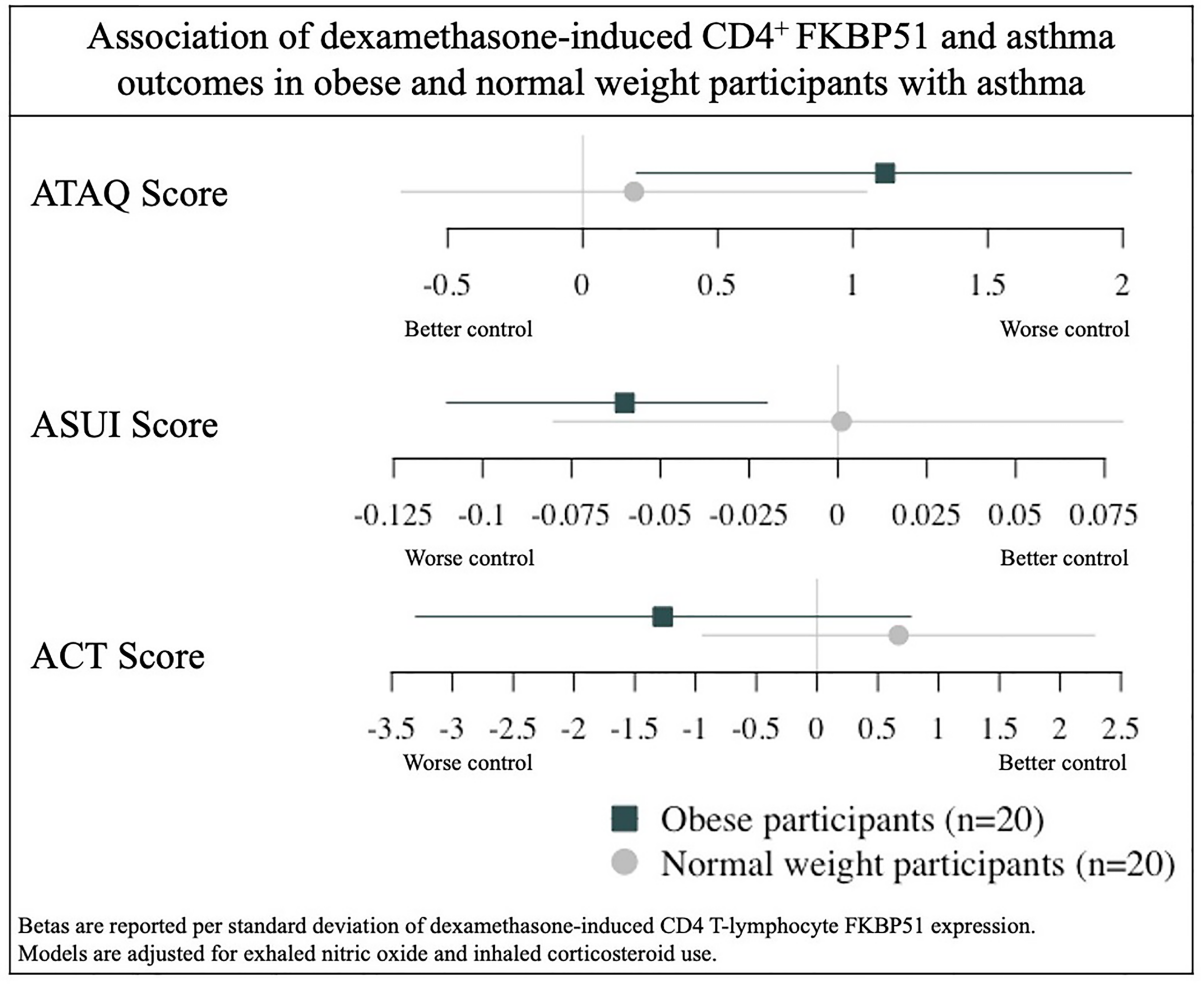
Association of dexamethasone-induced CD4+ FKBP51 and asthma outcomes in obese and normal weight participants with asthma.

In adjusted models including all 40 participants, baseline CD4^+^ Ki-67 (*p* = 0.07) or FKBP51 (*p* = 0.17) expression measured as geometric mean fluorescence intensity (MFI) was not associated with ATAQ score or any of the three secondary outcomes. In all participants, higher DEX-induced CD4^+^ T-lymphocyte FKBP51 expression was associated with a higher ATAQ score (β [95% CI] per SD: 0.71 [0.08, 1.34]). There were no associations with secondary outcomes of FEV1pp, ACT, or ASUI (**Table 2**). Obesity status (normal weight or obese) modified the association between DEX-induced CD4^+^ T-lymphocyte FKBP51 expression for both ATAQ score (*p*-interaction = 0.05) and ACT score (*p*-interaction = 0.07) but not for outcomes of ASUI score and FEV1pp (**Table 2**). Subgroup analyses demonstrated a statistically significant association of higher DEX-induced CD4^+^ T-lymphocyte FKBP51 expression with worse symptom scores (β [95% CI] per SD for ATAQ: 1.12 [0.20, 2.03]; ASUI: −0.06 [−0.11, −0.02]; ACT: −1.27 [−3.3, 0.77]). These associations and trends were not observed in normal weight participants (**Figure 1**). There was no association with worse FEV1pp in either group (**Table 2**).

**Table 2:** Beta (95% CI) per standard deviation of CD4^+^ Ki-67, FKBP51, and dexamethasone-induced FKBP51 expression to asthma control and lung function in obese and normal weight participants with asthma.

Outcome	All participants (*n* = 40)	Subgroup analysis		
		Obese participants (*n* = 20)	Normal weight participants (*n* = 20)	*p*-Interaction
**CD4^+^ Ki-67**				
ATAQ score	0.65 (−0.03, 1.33)	0.82 (−0.31, 1.95)	0.25 (−0.62, 1.12)	0.124
ACT score	0.12 (−1.18, 1.43)	−0.60 (−2.98, 1.77)	1.11 (−0.42, 2.63)	**0.062**
ASUI score	−0.01 (−0.05, 0.04)	−0.02 (−0.09, 0.04)	−0.00 (−0.08, 0.07)	0.900
FEV1pp	2.61 (−2.33, 7.55)	3.12 (−2.86, 9.09)	1.87 (−6.72, 10.46)	0.932
**CD4^+^ FKBP51**				
ATAQ score	0.45 (−0.18, 1.09)	0.40 (−0.75, 1.55)	0.21 (−0.60, 1.03)	0.392
ACT score	0.34 (−0.85, 1.54)	0.51 (−1.78, 2.81)	0.72 (−0.76, 2.20)	0.533
ASUI score	−0.01 (−0.06, 0.04)	−0.00 (−0.07, 0.06)	−0.02 (−0.10, 0.05)	0.687
FEV1pp	−2.02 (−6.58, 2.54)	−1.78 (−7.69, 4.12)	−4.13 (−11.98, 3.71)	0.705
**CD4^+^ dexamethasone-induced FKBP51**				
ATAQ score	**0.71 (0.08, 1.34)**	**1.12 (0.20, 2.03)**	0.19 (−0.67, 1.05)	**0.05**
ACT score	−0.36 (−1.60, 0.89)	−1.27 (−3.3, 0.77)	0.67 (−0.94, 2.28)	**0.07**
ASUI score	−0.03 (−0.07, 0.02)	−**0.06 (**−**0.11**, −**0.02)**	0.00 (−0.08, 0.08)	0.22
FEV1pp	−2.48 (−6.59, 1.63)	−0.91 (−6.39, 4.57)	−4.21 (−11.20, 2.78)	0.57

Models are adjusted for ICS and exhaled NO. The p-value shown is interaction representing effect modification of obesity status on the association of intracellular protein with the outcome of interest. Bolded values are statistically significant at p < 0.05 and p < 0.10 for interactions. Bold values denote statistical significance.

## Discussion

This study identifies increased prevalence of a distinct CD4^+^ subset-expressing FKBP51 and Ki-67 in obese children with asthma compared with a matched sample of normal weight children with asthma. Although baseline expression of FKBP51 and Ki-67 in CD4^+^ T-lymphocytes was not associated with asthma control, DEX-induced intracellular expression of FKBP51 from CD4^+^ cells was associated with worse asthma control. This association was stronger in the obese participants compared with normal weight, which means it may be uniquely contributing to asthma severity in obese children. The association of DEX-induced FKBP51 with the ATAQ score demonstrated an increase in ATAQ score of 1.12 per SD in obese participants which is greater than the MCID of 1 (17). The beta for the ASUI score was 0.06 which did not reach the MCID of 0.09 (18, 19) although the reported beta of 0.06 is per a single SD of CD4^+^ T-lymphocytes DEX-induced FKBP51 expression and therefore changes may be greater than the MCID across a wider variation of FKBP51 expression.

The expression of Ki-67—further supported by the high correlation between FKBP51 and Ki-67 expression—in these cells indicates a high proliferative activity. We speculate this may be related to the metabolic enhancing effects of FKBP51 (23). Proliferation of CD4^+^ T-lymphocytes has been observed in children with asthma (24) and proliferation of Th17 cells has been found to be increased in non-eosinophilic neutrophilic asthma (13). Although the proliferative activity (as assessed by Ki-67 expression) was not associated with morbidity, the higher proliferation of CD4^+^ T-lymphocytes in this matched cohort of obese children with asthma suggests it may play a role in the unique phenotype of obese children with asthma.

The expression of FKBP51 in peripheral blood cells is known to be induced by corticosteroids (9) and its overexpression can inhibit corticosteroid signaling, leading to resistance *via* a negative feedback loop (10, 11). Specifically, FKBP51 inhibits translocation of the glucocorticoid receptor (GR) into the nucleus and maintains the GR in a low-affinity state for steroid binding (25). FKBP51 can also drive inflammation *via* the NFκB pathway (26). Consistent with this, in a house dust mite animal model of asthma, FKBP51 overexpression decreased steroid sensitivity, while FKBP51 silencing improved steroid sensitivity (26). Although our findings are associative in nature, prior studies of FKBP51 silencing with improvement in steroid sensitivity in murine models (26) and increased expression of steroid-responsive genes in fibroblast cells (27) enhance the biologic plausibility for causality. In humans with asthma, higher total PBMC dexamethasone-induced FKBP51 transcriptional expression has been associated with less clinical improvement after ICS therapy (7), with similar findings of FKBP51 expression from epithelial cells (28). However, cellular source for FKBP51 as it relates to asthma severity has never been described and herein, we identify FKBP51 expression in CD4^+^ T-lymphocytes as a possible mediator of steroid resistance in obese asthma. The unique association of CD4^+^ T-lymphocyte FKBP51 expression with asthma morbidity among obese participants is consistent with CD4^+^ T-lymphocytes being central to the pathophysiology of asthma (29); however, molecular targets are currently limited to those in the Th2 pathway (30). The findings of this study suggest a non-Th2 mechanism of disease and provide insights into why obese individuals may be less responsive to steroids, given CD4^+^ T-lymphocytes that are less steroid responsive. It is conceivable that FKBP51 may represent a novel therapeutic target for these individuals; however, this is challenging given its intracellular location and the need for therapies to be specific to FKBP51 [and not affect FKBP52, for example, which enhances steroid sensitivity (27)]. The small molecule SAFit2 ligand, with high specificity for FKBP51, could represent a therapeutic target; reports have shown it to reduce the physiologic effects of FKBP51 in murine studies (31, 32).

Our study carries limitations which merit further investigation. Firstly, although we did our best to account for known confounders, there remain potentially unaccounted for environmental factors that may influence our findings. Future study in a more controlled preclinical animal model or *via in vitro* cell lines will help further our understanding of this pathway. High-dimensional flow cytometry evaluation explored a limited set of markers testing CD4^+^ activation, proliferation, and skewing profiles. There is some suggestion in our data the increased FKBP51 may be more pronounced among regulatory T cells (**Supplementary Figure S5**); in a separate study intratracheal administration of regulatory T cells has been shown to reduce airway inflammation and hyperresponsiveness when administered in a murine allergic asthma model (33). However, further phenotyping potentially with single-cell RNA sequencing to assess the transcriptome of these cells would provide unbiased transcriptomic signatures for CD4^+^ subpopulations. Although the cells were stimulated with dexamethasone, TCR sequencing and stimulation with a specific allergen such as dust mite could have provided additional insights into their functional atopic response. Our participants were 90% Black, which is a strength given the higher prevalence of asthma and obesity in this population (34, 35), but may limit the generalizability of our findings. Additionally, inclusion of healthy controls without asthma to assess FKBP51 expression would further contextualize our findings. Lastly, our findings represent a relatively small sample size; replication of the role of dexamethasone-induced CD4^+^ T-lymphocyte FKBP51 in a larger cohort would strengthen findings. However, unlike prior studies, our study design utilized matched participants to mitigate potential confounding by differences in disease severity and ICS use between obese and normal-weight participants with asthma. This may be the reason that previously described differences, such as Th1 inflammation in obese participants, were not observed in our study. Additional strengths of our study are that we targeted specific cellular sources of protein expression using flow cytometry and unsupervised clustering analysis, and we had extensive clinical phenotyping of our participants.

Our findings demonstrate that dexamethasone-induced CD4^+^ T-lymphocyte FKBP51 expression is associated with worse asthma control in obese children with asthma and may contribute to the unique steroid-resistance observed in this population. This suggests CD4^+^ T-lymphocyte FKBP51 expression may represent a novel therapeutic target of particular relevance in obese individuals with asthma.

## Data Availability Statement

The raw data supporting the conclusions of this article will be made available by the authors, without undue reservation.

## Ethics Statement

The studies involving human participants were reviewed and approved by Johns Hopkins Institutional Review Board (IRB00074171). Written informed consent to participate in this study was provided by the participants’ legal guardian/next of kin.

## Author Contributions

VT, KS, MD, EB, MM, and FD conceived the presented idea. VT, AM, NX, and FRD performed flow cytomety and processed flow cytometry data. VT, KS, HW, NH, and MM performed biostatistical analysis. All authors contributed to the article and approved the submitted version.

## Funding

This work was directly funded by a CHEST Foundation Research Grant for Severe Asthma, a Baurenschmidt Award from the Johns Hopkins Eudowood Foundation, NIH NIEHS P50ES018176-09, “AIRWEIGHS”, and EPA (agreement number 83615201 to Hansel). VT is supported by a National Institutes of Health (NIH) T32 (T32HL007534-36) and F32 (NHLBI 1F32HL149258-01) grant, AM is supported by an NIEHS T32 grant (T32ES07141), KS is supported by an NIH K08 (NHLBI K08HL13205), EB is supported by an NIH K23 (NIEHS K23ES029105-01A1), MD through a K01 (OD K01OD019918), and FRD by NIH R01 (NHLBI HL131812).

## Conflict of Interest

MM reports royalties from Uptodate, consulting fees from Glaxo Smith Kline and Celgene, outside the submitted work. NH reports grants from COPD Foundation, grants and personal fees from AstraZeneca, grants and personal fees from GSK, grants from Boehringer Ingelheim, and personal fees from Mylan during the conduct of the study, outside the submitted work.

The remaining authors declare that the research was conducted in the absence of any commercial or financial relationships that could be construed as a potential conflict of interest.

## Publisher’s Note

All claims expressed in this article are solely those of the authors and do not necessarily represent those of their affiliated organizations, or those of the publisher, the editors and the reviewers. Any product that may be evaluated in this article, or claim that may be made by its manufacturer, is not guaranteed or endorsed by the publisher.
