# Supplementary material for: Dexamethasone-Induced FKBP51 Expression in CD4+ T-Lymphocytes Is Uniquely Associated With Worse Asthma Control in Obese Children With Asthma
Source: Front Immunol. 2021 Oct 15;12:744782. doi: 10.3389/fimmu.2021.744782 (PMC8554235; doi:10.3389/fimmu.2021.744782)

**Dexamethasone-induced FKBP51 expression in CD4^+^ T-lymphocytes is uniquely associated with worse asthma control in obese children with asthma**

**Supplementary Methods**

**Figure E1. Example of FKBP51 upregulation after incubation in dexamethasone 10^-6^ for 24 hours**

**Table E1. Antibody panel for lymphocyte characterization**

| **Lymphocyte** | ANTIBODY | FLOUROCHROME | CLONE | COMPANY | CATALOG# |  |
| --- | --- | --- | --- | --- | --- | --- |
|  |  |  |  |  |  |  |
| Blue Laser 488nm | **CD3** | BB515 | UCHT1 | BD | 564465 | Surface |
|  | **FoxP3** | Per-CP-Cy5.5 | PCH101 | ThermoFisher | 45-4776-42 | Intracellular |
|  | **IL-4** | PE-Cy7 | MP4-25D2 | BD | 560672 | Intracellular |
|  | **IL-9** | PE-CF594 | MH9A3 | BD | 564255 | Intracellular |
|  |  |  |  |  |  |  |
| Red Laser 635nm | **FKBP51** | Ax647 | epr6617 | Abcam | ab198979 | Intracellular |
|  | **CD8a** | APC/fire 750 | HIT8a | Biolegend | 300932 | Surface |
|  | **IL-17A** | APC-R700 | N49-653 | BD | 565163 | Intracellular |
|  |  |  |  |  |  |  |
| Violet Laser 405nm | **TNF-a** | BV605 | Mab11 | BD | 563915 | Intracellular |
|  | **CD127** | BV421 | HIL-7R-M21 | BD Biosciences | 562436 | Surface |
|  | **CD19** | BV510 | SJ25C1 | BD Biosciences | 562947 | Surface |
|  | **IL-10** | BV650 | JES3-9D7 | BD | 564051 | Intracellular |
|  | **CD25** | BV711 | BC96 | BioLegend | 302636 | Surface |
|  | **Ki-67** | BV786 | B56 | BD | 563756 | Intracellular |
|  | **IFN-gamma** | BV750 | B27 | BD | 566357 | Intracellular |
|  |  |  |  |  |  |  |
| UV laser 355nm | **Live/Dead** |  |  | Invitrogen | L23105 |  |
|  | **CD4** | BUV395 | SK3 | BD | 563550 | Surface |
|  | **CD14** | BUV737 | M5E2 | BD | 612763 | Surface |

**Figure E2. Gating Strategy:** Single cells were identified followed by live-dead discrimination. Live cells were excluded for CD14^+^ populations and inclusion of CD3^+^ populations to remove monocytes and identify T-lymphocytes, respectively. CD4^+^ and CD8^+^ populations were identified. Within the CD4^+^ population, Th1 cells were defined as IFN-γ^+^, Th2 as IL-4^+^, Th9 as IL-9^+^ and Th17 as IL-17^+^. Regulatory T-cells were defined as CD4^+^CD25^+^CD127^-^FoxP3^+^ (not shown).

**
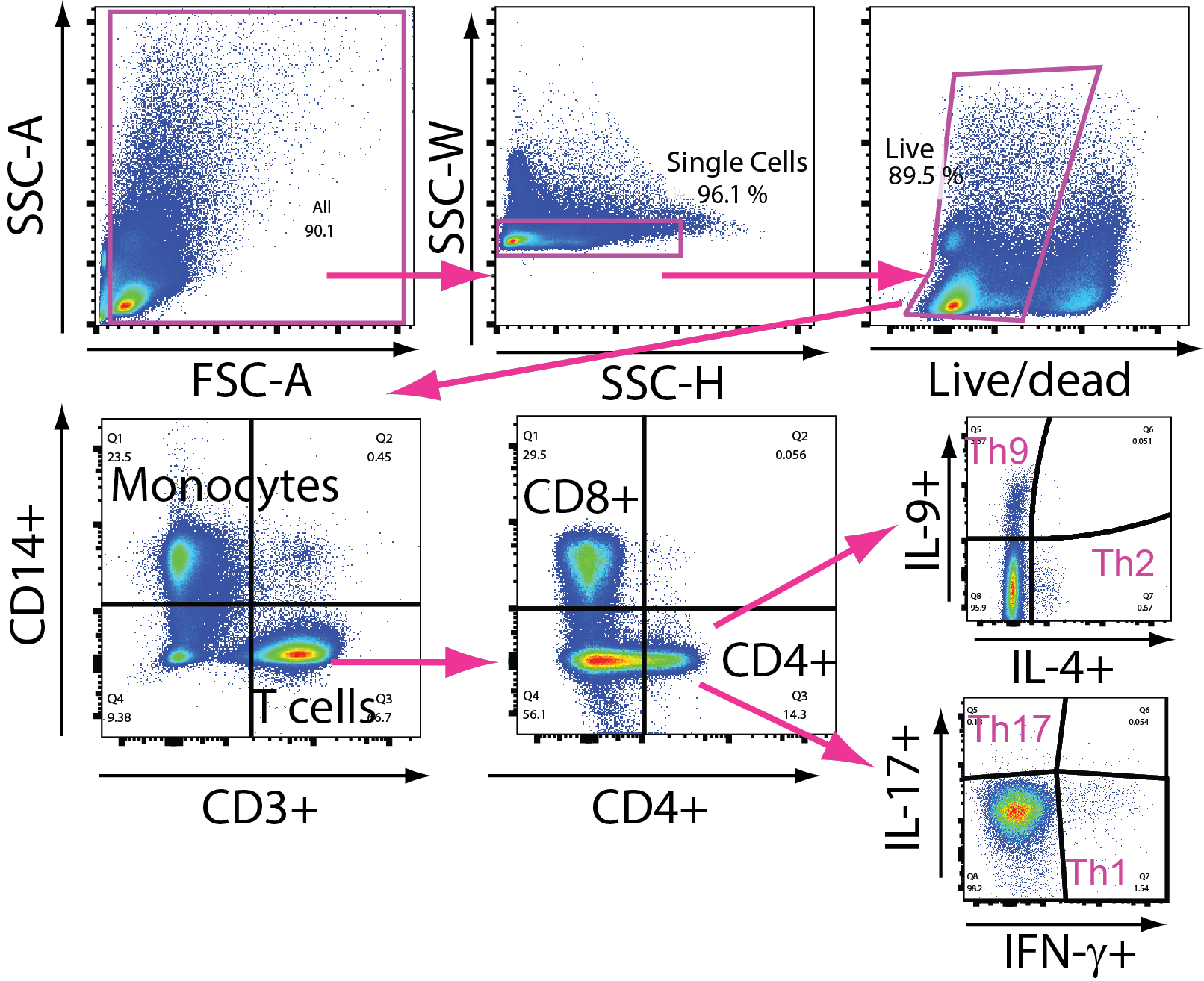
**

**Figure E3. Differential cluster map shows clusters where number of cells were increased by 100% in obese participants with asthma compared to normal weight participants with asthma**

Differential cluster map shows clusters where the number of cells within the cluster were increased by 100% in obese participants (red, n = 3). Cytokine profile (inset) showing MFI in the selected clusters.

**Figure E4. Expression of FKBP51 in CD4^+^ T-lymphocytes in obese compared to non-obese individuals with asthma**

**
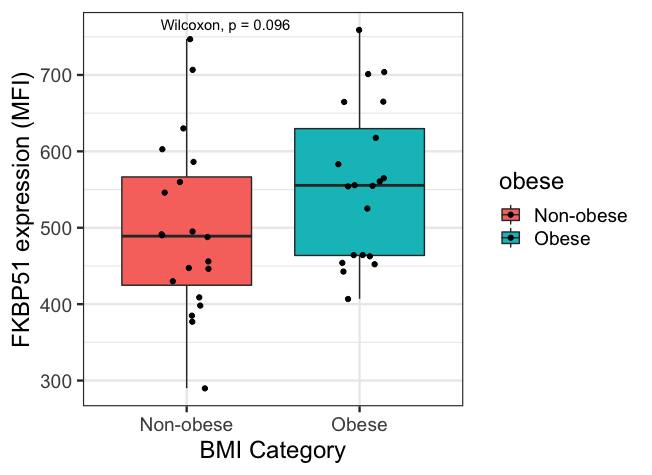
**

**Figure E5. Expression of FKBP51 in CD4^+^ T-lymphocyte subpopulations in obese compared to non-obese individuals with asthma**

**
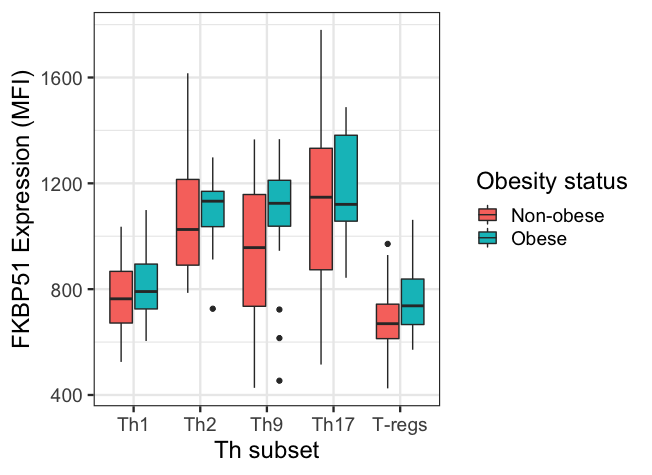
**

**Figure E6. Differential cluster map after dexamethasone-incubation shows clusters where number of cells were increased by 100% in obese participants with asthma compared to normal weight participants with asthma**

Differential cluster map shows clusters where the number of cells within the cluster were increased by 100% in obese participants (red, n = 2). Cytokine profile (inset) showing MFI in the selected clusters.

**Figure E7. Expression of DEX-induced FKBP51 in CD4^+^ T-lymphocytes in obese compared to non-obese individuals with asthma**

**
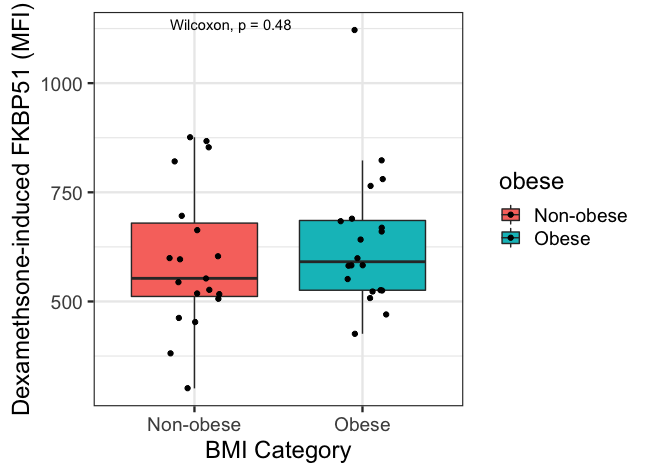
**

**Figure E8. Expression of DEX-induced FKBP51 in CD4^+^ T-lymphocyte subpopulations in obese compared to non-obese individuals with asthma**


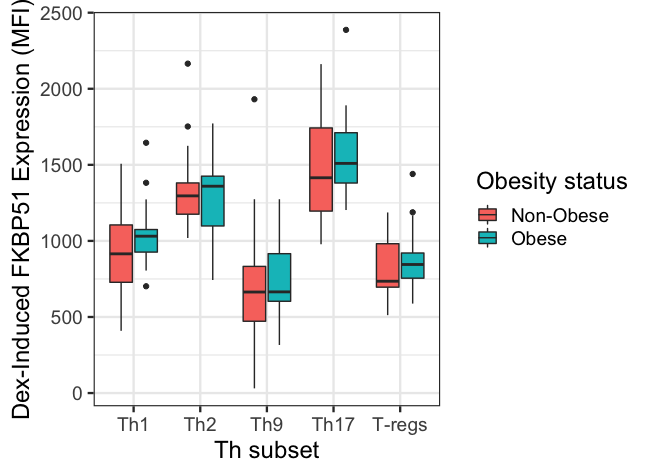

Supplement: Supplementary file 1 [file DataSheet_1.docx]
